# Diagnostic yield and therapeutic outcome of hysteroscopy in women with infertility in a referral clinical setting: a Port Harcourt, Nigeria experience

**DOI:** 10.11604/pamj.2021.38.155.27101

**Published:** 2021-02-11

**Authors:** Emeka Ray-Offor, Tamunomie Kennedy Nyengidiki

**Affiliations:** 1 Oak Endoscopy Centre, Port Harcourt, Rivers State, Nigeria,; 2 Department of Surgery, University of Port Harcourt Teaching Hospital, Rivers State, Nigeria,; 3 Department of Obstetrics and Gynaecology, University of Port Harcourt Teaching Hospital, Rivers State, Nigeria

**Keywords:** Hysteroscopy, infertility, outcome

## Abstract

**Introduction:**

intrauterine pathologies were traditionally evaluated by blind dilatation and curettage along with hysterosalpingography. Hysteroscopy is a veritable tool for evaluation of uterine cavity pathologies with an increasing availability in Nigeria. The study aims to report the diagnostic yield and therapeutic outcome of hysteroscopy in women with infertility from a Nigerian metropolis.

**Methods:**

a retrospective cross-sectional study of consecutive cases of infertile women referred for hysteroscopy to an ambulatory care endoscopy facility in Port Harcourt, Nigeria. The variables collated included age, parity, past gynaecological history, indication, hysteroscopy findings and interventions. Statistical analysis was performed using SPSS version 25.

**Results:**

included in study were 75 patients undergoing a total of 124 hysteroscopic procedures. The age range of patients was 25 to 56 years (mean 40.7 ± 5.9 years). A positive diagnostic yield of 93.3% with non-visualization of intracavitary uterine pathology in 5 patients was recorded. The most common pathologies were submucous myoma-25(31.3%), endometrial polyps- 18(22.5%) and intrauterine adhesions-18(22.5%). There was no statistical difference between the mean age of patients with or without polyp, myoma and intrauterine adhesions (P = 0.185, P = 0.510 and P = 0.619 respectively) but a non-linear relationship between age and polyp detection (Eta2 = 0.024). Operative hysteroscopies were all performed on out- patient basis but staged in 30(40.0%) patients.

**Conclusion:**

benign uterine growths and intrauterine adhesions are frequent causes of uterine factor infertility. A high successful completion rate of hysteroscopic interventions was achieved.

## Introduction

Infertility is a disease of the reproductive system defined by the failure to achieve a clinical pregnancy after 12 months or more of regular unprotected sexual intercourse [1]. Globally, it is estimated that 72.4 million women are infertile and that 40.5 million of these women are currently seeking fertility treatment [1]. In modern times before undertaking advanced assisted reproductive procedures of in vitro fertilization and embryonal transplant, a base line hysteroscopy is recommended especially in failed previous attempts [2]. Hysteroscopy is a sensitive tool for diagnosis and treatment of intrauterine pathologies. Generally, the indications for performing hysteroscopy in women include uterine bleeding disorders, Müllerian tract anomalies, retained intrauterine contraceptives or other foreign bodies, retained products of conception, desire for sterilization, recurrent miscarriage, and infertility [3].

Pantaleoni is credited with performing the first hysteroscopy in 1869 but over the years the practice has evolved with improvements in optics and miniaturization of endoscopes and ancillary equipment [4]. Traditionally, evaluation of intrauterine pathology was by blind dilatation and curettage along with hysterosalpingography [5]. There is improved diagnosis with the introduction of transvaginal ultrasound however focal lesions can be missed especially in cases of two or more lesions [6]. Both diagnostic and operative hysteroscopy require uterine distension to effectively visualize the uterine cavity. A selection of distension medium by the surgeon is dependent on the patient´s condition, procedure to be performed, and the electrosurgical device to be used [7]. The options for uterine distension include insufflation with carbon dioxide (CO_2_) gas, or the instillation with electrolytic or non-electrolytic liquid distention media.

Hysteroscopy can be performed as an outpatient (office) procedure or as an inpatient, hospital-based service with monitored regional or general anesthesia care. The gynaecologic endoscopy practice of hysteroscopy is less intrusive, cost effective and often performed as day care surgery [8]. Ambulatory hysteroscopy is an out-patient -based hysteroscopy procedure, performed in an adapted hospital setting or a dedicated facility where, ideally, patient remains conscious and walks in and out of the unit without the need for prolonged post-operative recuperation or monitoring [9]. With advancement in technology hysteroscopic procedure are largely done without anaesthesia with the introduction of miniature scopes; hence outpatient office hysteroscopy. In Nigeria, hysteroscopy service is increasingly available in assisted reproductive technology centers and a few private and public hospitals [10-12]. However, there is paucity of literature on treatment outcome including pregnancy rates following therapeutic hysteroscopic intervention in infertile females from parts of the country. This study aims to report the diagnostic yield and therapeutic outcome of hysteroscopy among infertile female Nigerian patients from a centre in Niger Delta region of Nigeria.

## Methods

This was a retrospective cross-sectional study of women that were referred to and underwent hysteroscopy at a multi-disciplinary endoscopy facility located in Port Harcourt metropolis, Rivers state, Nigeria from June 2014 to December 2019. The Centre receives referrals from within Rivers State and nearby states of the Niger delta region of Nigeria. The study population comprised all consecutive women with infertility that underwent hysteroscopy in the centre and excluded were patients with other known indications for hysteroscopy and patients less than 20 years of age. An access to Centre records for relevant data was sought with a study proposal submitted to the Ethical Committee of the Centre, which was approved. A pre-structured Microsoft Excel spreadsheet was used to collate data on study patients. The variables collated included age, parity, past gynaecological history, indication, hysteroscopy findings and interventional outcome- number of interventions for pathologies, successful completion rate and adverse effect.

An informed consent was obtained according to Helsinki declaration. A careful clinical evaluation for comorbidities and pelvic examination including saline hysterosonography performed by a gynae-sonologist at a referral hospital or transvaginal sonography performed by a radiologist in study centre. Other relevant investigations were done. The hysteroscopy procedure was scheduled in the immediate period following menstruation (commonly on 10^th^ day of menstrual cycle). There was routine insertion of 400µg misoprostol into the posterior vaginal fornix the day preceding procedure for ripening of cervix (only in patients for which a resectoscope was used). The equipment used was Karl Storz (Germany) Camera unit, 150W Xenon light source, HD monitor, AIDA Image capture device, Bettocchi hysteroscope with a 300 rod lens telescope, (Karl Storz GbmH & Co., Tutlingen, Germany), Automated infusion pump (C-Fusor 1000 Mx4810 by Smiths Medicals) and 300W Electrosurgical Unit.

For diagnostic hysteroscopy, sedation/analgesia protocol was conscious sedation using benzodiazepine and an opioid analgesic (pentazocine 30mg) with intracervical infiltration of 2mls of 1% Lidocaine. Regional (spinal) anaesthesia was the preferred choice for operative hysteroscopy. General anaesthesia was offered to patients who objected to regional anesthesia after due counselling. With patient in lithotomy position, for diagnostic procedures a “non-touch” technique was used to gain access into the uterine cavity while for operative procedures a Sims speculum was inserted into the vagina and anterior cervix grasped with tenaculum. The cervix was dilatated using Hegar´s dilator size 6 to admit a 5mm resectoscope usage (if necessary). Normal saline was the distension medium for diagnostic procedures and instilled at a controlled pressure of 100-150mm Hg. Complete visualization of the endometrial cavity was done before any operative procedure was undertaken. The distension medium of choice for operative procedure was 1.5% glycine with resection performed using a monopolar energy. Fluid deficit was estimated manually after the procedure. All patients were monitored until full recovery and discharged home on the same day with prescription of doxycycline capsules, metronidazole, and analgesics for operative cases. In cases of adhesiolysis, a 6Fr catheter was inserted into the uterus at the end of procedure, then removed on the 7^th^ day post procedure. Estradiol valerate 2mg thrice daily for 21 days and medroxyprogesterone acetate 5mg from the 22^nd^ day was given for seven days for three cycles. Second look hysteroscopies were performed for some patients to certify resolution of prior pathologies in the next cycle. Data analysis was performed using IBM SPSS Statistics for Windows, Version 25 Armonk, NY, USA. A univariate analysis was performed for frequencies with continuous variables presented as mean + standard deviation and percentages while categorical variables as simple percentages. For multivariate analysis, a relationship between cases of with pathologies detected and age of patients was determined with ANOVA. A statistical significance was set at <0.05.

## Results

**Figure 1:**
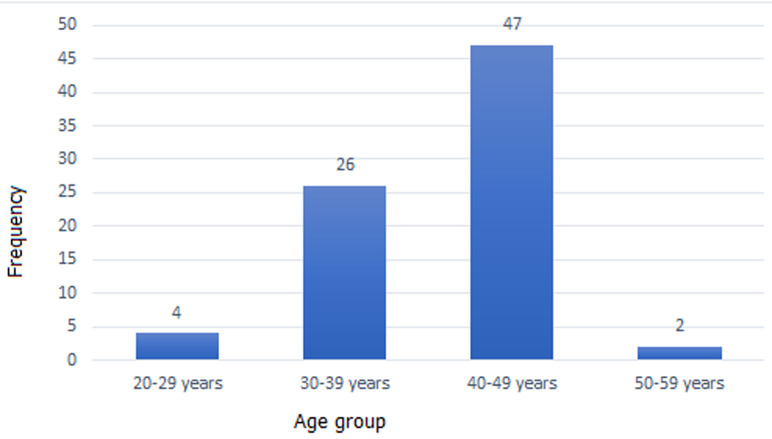
age distribution of study population

A total of 124 hysteroscopic procedures were performed in 75 women with infertility and included in the study. The age range was 25 to 56 years (mean 40.7 ± 5.9). The highest frequency of hysteroscopy was performed for patients in the 5^th^ decade of life, 45(60.0%) (Figure 1). Majority of patients were literate with 58(77.3%) having a post-secondary/tertiary education (Table 1). There was a high rate of previous uterine surgeries in the patients studied: termination of pregnancy- 37(49.3%); myomectomy-26(34.7%); and prior adhesiolysis- 6(8.0%). Also, there was a history of previously failed in vitro fertilization in 14(18.7%) patients. At least one pathology was detected in all but 5 patients resulting in a positive diagnostic yield of 93.3%. More than one pathology was noted in 5(6.7%) cases. The most common findings were submucous myoma 26(31.3%), polyps 18(22.5%), and intra intrauterine adhesions-18(22.5%) (Table 2). There was no statistical difference in the mean age of patients with polyp, myoma nor adhesion (P=0.185, P=0.510 and P=0.619 respectively) (Table 3) A non-linear relationship was noted between age and polyp detection (Eta2 = 0.024). Operative procedures performed were hysteroscopic myomectomy-31(25.0%); adhesiolysis-30(24.2%), endometrial polypectomy- 16(12.9%), fetal bone extraction and evacuation of retained products of conception-4(3.2%). Also, foreign body (sutures) were extracted in 2 cases (1.6%). Operative hysteroscopic procedures were staged in 30(40.0%) patients for type I and II myomas including a follow-up evaluation for operative hysteroscopy in 12 cases. All procedures were performed on out- patient basis. There was a uterine perforation and another failure to access the uterine cavity because of severe cervical stenosis.

**Table 1:** socio-demographic of women in study population

Variables	Value (n=75)	Percentage
Age (mean)	40.7 ± 5.9 years	-
Level of education		
Primary	1	1.3
Secondary	2	2.7
Post-secondary	58	77.3
Not stated	14	18.7
Indication		
1° infertility	15	2.1
2° infertility	60	77.8
Previous uterine surgeries		
Myomectomy	26	34.7
Dilatation and curettage (termination of pregnancy)	37	49.3
Adhesiolysis	6	8.0
History of in-vitro fertilization	14	18.7

**Table 2:** hysteroscopic findings and interventions

Hysteroscopic findings (n=75)	Frequency (%)
Submucous myoma	25 (31.3)
Polyps	18 (22.5)
Intrauterine adhesions	18 (22.5)
No pathology detected	5 (6.3)
Retained products of conception	3 (3.8)
Haematometra	3 (3.8)
Scar tissue	2 (2.5)
Foreign bodies (sutures)	2 (2.5)
Mullerian duct anomalies	1 (1.2)
Fetal bones	1 (1.2)
Endometritis	1 (1.2)
Hypoplastic uterus (Sheehan syndrome)	1 (1.2)
**Total**	**80 (100)**

**Table 3:** comparison of age distribution of patients with major pathologies detected at hysteroscopy

Pathology	Pathology present	Mean age (years)	Pathology absent	Mean age (years)	P value
Polyp	18	42.3 ± 7.1	57	40.2 ± 5.4	0.185
Myoma	25	41.4 ± 4.8	50	40.4 ± 6.4	0.510
Adhesion	18	40.1 ± 6.1	57	40.9 ± 5.9	0.619

## Discussion

Diagnostic and some operative hysteroscopy procedures can be done in a clinic setting using local anaesthesia and fluid distension media, while more complex procedures are generally performed as day surgery under general anaesthesia [13]. In this study of infertile female Nigerian patients, all diagnostic and operative hysteroscopies were performed as day-case procedures with satisfactory outcome. There was a high diagnostic yield among patients. This is high when compared to reports of positive detection rates during hysteroscopy of 45% to 72.9% from different parts of Nigeria [11-15]. The high diagnostic yield of hysteroscopy in this study can be partially explained by the referral bias following several attempts at achieving a pregnancy in addition to findings of abnormalities from other radiological methods of endometrial evaluation.

Major uterine cavity abnormalities were found in women seeking treatment for infertility with the most common intracavitary uterine pathology being submucous myoma in 25(31.3%) patients. Submucous myomas are noted to interfere with endometrial lining, inhibiting implantation and subsequently gestational failure. The distribution of pathology in this study is unlike other hysteroscopy studies from Nigeria that report a higher prevalence of intrauterine adhesion than myoma in infertile women but similar to a report from Nairobi, Kenya. Ten (10),11,13,14,15 endometrial polyps are localized and stalked hyperplastic overgrowths of glands and stroma that form a projection above the uterine surface and rarely include foci of neoplastic growth [16]. Eighteen (22.5%) patients had at least a polyp detected during hysteroscopy. There was no histopathologic diagnosis of neoplastic growth recorded from resected lesions. Intrauterine adhesions were detected in 18(22.5%) patients. These fibrous strands connecting parts of the uterine wall are commonly caused by inflammation or iatrogenic tissue damage and correspond to the high rate of previous history of uterine surgeries in the study population. Of note was a case of previous myomectomy with multiple suture strands seen traversing a narrowed uterine cavity. The cavity ballooned out on severing suture strands during hysteroscopy to reveal a submucous myoma which was resected.

A selection of distension media by the surgeon is dependent on the patient´s condition, procedure to be performed, and the electrosurgical device to be used [6]. Normal saline, a physiologic electrolytic solution, was the fluid of choice for uterine distention during diagnostic hysteroscopies and can be used when using bipolar energy with minimal endometrial damage. However, in this study, glycine- a non-electrolytic solution was used in resections of myomas and endometrial polyps. Occasionally, multiple procedures are often required to achieve satisfactory resection of large myoma about 5cm in diameter and this was also influenced by the location of the myoma or to achieve satisfactory anatomic results in the treatment of intrauterine adhesions. Hysteroscopic adhesiolysis was performed using cold scissors or bipolar electrosurgery to restore the size and shape of the endometrial cavity. Postoperative mechanical distention of the endometrial cavity and hormonal treatment facilitate endometrial regrowth appear to decrease the high rate of adhesion reformation [17].

Observational studies have demonstrated improvement in the spontaneous pregnancy rate after the hysteroscopic removal of the abnormality [18]. However, there is high level evidence that suggests the chance for pregnancy is significantly lower in infertile women with submucous myoma compared to other causes of infertility [19,20]. A significant number of operative hysteroscopies in the study population were for hysteroscopic myomectomy, this may have affected the post procedure conception rate. Randomized controlled trial however, has not shown that hysteroscopic removal of endometrial polyps, submucous fibroids, septa or intrauterine adhesions is likely to benefit women with otherwise unexplained infertility associated with these suspected uterine pathologies compared to a control intervention [21]. From follow-up calls, an overall conception and live birth rate of 36.4% was recorded with 3(9.1%) spontaneous pregnancies and 9(27.3%) intrauterine insemination-assisted pregnancies from 33 respondents who were previously having fertility challenges before the hysteroscopic procedures. Some patients were non-responsive in the follow-up phone calls. A limitation of this study is possible detection bias as the study population of infertile women mostly had hysteroscopy following radiological suspicion of intra-cavitary uterine pathology. This in addition to the small sample size, may not accurately reflect the population distribution of pathology. Albeit the study demonstrates the usefulness of hysteroscopy in pre-IVF work-up of infertile women.

## Conclusion

Benign uterine growths and intrauterine adhesions are frequent findings in infertile women in our environment. Hysteroscopy remains the mainstay in evaluation of endometrial factor infertility. The screening of patients with transvaginal sonography/saline sonohysterography may increase the diagnostic yield of hysteroscopy. A high rate of successful completion of hysteroscopic interventions was achieved.

### What is known about this topic

- Hysteroscopy is the gold standard for evaluation of intracavitary uterine pathologies;
- Hysteroscopy has both diagnostic and therapeutic advantages over radiological investigations of transvaginal ultrasonography, saline hysterosonography and hysterosalpingography;
- There is increasing report of the utilization of hysteroscopy by assisted reproductive centres and tertiary hospitals in Africa but paucity of fertility outcome following therapeutic interventions.

### What this study adds

- Benign uterine growths and intrauterine adhesions are frequent findings in infertile women;
- Hysteroscopic interventions improved the pregnancy rates among the population of women studied following assisted conception or spontaneous pregnancy;
- This study reinforces the safe utilization of hysteroscopy in the evaluation and treatment of intracavitary pathologies.
